# Address

**Published:** 1875-04

**Authors:** J. Richardson


### DENTAL REGISTER.

VOL. XXIX.] APRIL, 1875. [No. 4.

### ADDRESS.

BY J. RICHARDSON, M. D., D. D. S.

*At the Thirtieth Annual Commencement of the Ohio Dental College, March 4th,* 1875.

*Gentlemen Graduates:*—The agreeable duty has been assigned me this evening of felicitating you upon the completion of your collegiate course, and of assisting in the closing exercises of the session by offering to you such parting words as may seem appropriate on the occasion of your formal entrance upon the duties of your chosen profession. It has been quite a number of years since it has been my privilege to participate personally in the Commencement Exercises of this honored institution, and I am by no means insensible to the compliment implied in the invitation to do so now. To those who feel personally identified with the school, by reason of their relations as alumni, there attaches to these annual exercises an interest superadded to that which all must feel in the cause of dental education, *per se*.

Twenty-two years ago I assembled day after day with my class mates in this amphitheater, and at the close of the session passed out as you will do to-night endorsed by the faculty as qualified to take upon myself the important duties and obligations of my calling. Whatever measure of success may have attended my professional life since that time, rests, I am sure, upon the fundamental and underlying truths and principles taught me in this place. Later, I became more nearly identified with the interests of the school by association with the faculty. After a few years this connection ceased, since which time I have led a life of comparative professional isolation, but carrying with me always the old and unabated love for my Alma Mater. Back once more within the old walls, and charged with the duty of addressing you for a brief time, I enter upon its discharge by tendering you first, as was tendered to myself and others upon a similar occasion, a formal but most friendly and cordial welcome to the ranks of the profession.

I shall depart this evening, gentlemen, from the usual and time-honored custom of imparting words of advice to you upon matters pertaining to your future personal and professional deportment, and the varied personal duties and obligations which will grow out of your new relations to the profession and the public. I shall assume that you are entering upon the life-work before you with adequate and intelligent views of all these things. In the brief time allotted me, I prefer rather to talk to you about some matters of general concernment to to the profession at large, and therefore of interest, I hope, to you, since they relate to a calling which you have this evening formally espoused, and with whose interests you are now intimately identified. I shall call your attention therefore to some thoughts upon the nature and requirements of dental practice, with perhaps such supplementary reflections as the subject may suggest.

When we survey the entire field of dental practice in the light of its highest requirements, we can not fail to be impressed with the diversity and, I may say, the antipodal nature of its demands upon us. It will require but a moment's reflection to enable any one to perceive a broad and well defined line of distinction between the essential nature and corresponding requirements of dental practice and that of other departments of general medicine, and that this distinction is so pronounced as to render the former exceptional and anomolous. Invading the domains of medical science on the one hand, it levies unsparing contributions upon many of its most important departments, while on the other, it stretches far out into the realms of art, demanding even more inexorably still, tribute of its multitudinous processes and appliances, manual and mechanical.

Dentistry, viewed in its scientific aspect, contemplates an extended range of studies wholly worthy of the best exercises of the mind, and which can not be ignored by any one who aspires to completeness of qualification for the high and responsible duties of his calling. A very limited survey of the studies contemplated in the cultivation of the science of dentistry will serve to indicate their nature and scope.

The intimate relations of the teeth and fifth pair of nerves is, in itself, not only a study of curious interest, but one of prime importance to us as dental practitioners. Besides the many local sympathetic affections about the head and face which, by reason of their immediate association with the trifacial nerve, are clearly traceable, in many instances, to dental disorders or their sequellæ, acting as primary sources of irritation, there are, as shown by reports of well authenticated cases, still others of graver import, affecting remote organs, having a similar origin. "Almost inappreciable," says a well-known author, "are the beginings of many fatal diseases, and could the grave reveal its secrets, I have not a doubt, when I consider the number of diseases produced by disordered teeth that it would be found that thousands are there in whom the first fatal impulse was given by a diseased state of these organs." Here are thoughts worthy of our highest consideration, and the author's views will be fully justified when we come to reflect that the nervous system furnishes the conditions, either immediate or remote, under which the organic functions are performed, and that the fifth pair, from which the teeth are supplied, are so intimately connected, through its organic filaments, with the sympathetic system, as, according to Dr. Carpenter, to supply in the head the place of a separate ganglionic system. In the light of these anatomical and physiological facts, what an inviting field is here afforded the dental student for research and inquiry into the possible agencies which the diseases of the dental organs and their associate parts may have in perverting the functions of remote and vital organs of the body, and in the consequent production of some of the gravest and most formidable diseases known to pathology.

Not less inviting or less important are the studies which relate to the constitutional disturbances oftentimes incident to teething, and the influence of certain diseases peculiar to infancy and childhood, as exciting and predisposing causes of dental abnormities; the agency of modified nutrition in promoting functional derangement and structural impairment of the mother's teeth during gestation and lactation; the value of therapeutic and hygienic treatment in respect to improved structural development of the teeth; the hereditary transmission of structural defects and mal-arrangements of the teeth, abnormities in the conformation of the the jaws, and solutions of continuity in the hard and soft palates; the association of congenital idiocy with aberations in the configuration of the maxillary bones; the periodical character and malarial origin of many distressing forms of functional and sympathetic disorders of the dental organs; the many structural lesions connected with the oral cavity, as cancrum oris, necrosis, morbid growths, destructive and malignant affections of scrofulous and venereal origin, as well as the more benign pathological conditions so frequently found implicating the pulp and membranes of the teeth, the tonsils, glands, nasal passages and maxillary sinuses.

This brief summary of subjects of study coming fairly within the province of the dental practitioner, is not by any means intended to embrace every thing of professional interest or importance to us, but is suggestive rather of the general scope of medical acquirements contemplated in the scientific practice of dentistry. Certainly it comprehends enough to vindicate its claims to rank as a legitimate department of medicine.

I turn now for a few moments to consider it in its aspect of a mechanical art. I have said that there is a broad and fundamental line of demarkation between the essential nature of dental practice and that appertaining to the other departments of the healing art, and that this is so pronounced as to render the former phenomenal. It is well to bear this distinction in mind. To lose sight of it, is to breed false and mischievous notions of the true and inexorable demands and obligations imposed upon us as dentists.

Dentistry affords the only example within the entire range of medical practice where the treatment of important organs consists chiefly and essentially in the employment of manual processes. The anomolous character of operative practice grows out of the anomolous nature of dental caries. There is no department of dental practice held in higher esteem than that which relates to the conservative treatment of the natural teeth and measured by the frequency of its demands upon us, and the benefits conferred, it may fairly take precedence of all other departments of practice. Probably nine-tenths of all the cases presented for treatment relate to the operation of filling. Now, caries of the teeth is, in all its essential features, wholly unlike that affecting other portions of the osseous system, and demands for its remedial treatment modes of procedure quite as dissimilar. While the one is amenable to medical and surgical treatment and susceptible of reparation by the deposition and organization of new material, the other is irreparable except by the mechanical operation of filling Caries of the teeth, in its uncomplicated form, though an abnormal condition, can scarcely, in any proper sense, be considered a diseased or pathological one, but is simply a breach of continuity in the structure of tooth bone induced by chemical solvents, and, when not associated with exposure of the pulp, one of the most simple and benign of all structural lesions. A very large majority of all the cases submitted to us for treatment are of this uncomplicated kind, and it will scarcely be claimed that in respect to this class of cases, the operation of filling necessarily involves any more knowledge of the principles of medicine than some acquaintance with the structural characteristics of the teeth, and the anatomical relation of the pulp-chamber to the body of the tooth, that any one, whatever may be his medical attainments, who has proper perceptions of the simple mechanical principles involved in the preparation of the cavity, and is familiar with the working properties of the materials used, and expert in the use of needed instruments, may perform it as well as another. In the practice, therefore, of this most important, perhaps, of all departments, the application of the principles of medicine are clearly subordinated to the demands of manual or mechanical processes.

The differential characteristics of practice under consideration are still more sharply defined in what is called the mechanical department or, more properly, dental prosthesis. The construction of artificial sets of teeth involves, in a less degree even than filling, the application of the principles of medicine, embracing little more than a knowledge of the anatomy of the jaws and associated parts, and the treatment of some of the simpler forms of diseased action sometimes incident to the wearing of artificial substitutes, while the processes concerned in their fabrication are largely manipulative and mechanical. The same remarks are equally applicable to dental obturators and artificial palates, and to the various appliances for the correction of dental irregularities. Manipulation and mechanism are the predominant and characteristic features entering into all these various practices, and we find here, as in the operative department, the same subordination of medical acquirements to manual and mechanical processes.

I have not considered the high order of manipulative skill which the right practice of these several branches imperatively demands, or their absolute or relative importance as departments of dentistry, since they do not enter into the question I wish to present this evening.

If now we contrast the operative and mechanical branches of dentistry with the practice of either general, ophthalmic, or aural surgery, we shall find that no such subordination of medical acquirements to manual and mechanical processes exists in respect to the latter as have been clearly shown to obtain in reference to the former.

The practice of general surgery, though largely manipulative, is, not like operations at the chair or in the laboratory, *constructive* in its character, or, in other words, is not *productive.*

Whatever manipulative or mechanical appliances are employed in the practice of general surgery, it is difficult to conceive of any operation which is purely manual or mechanical, or that does not involve, in some measure, medical treatment, local or constitutional, before or after the operation, or both. Surgical practice has always intimate, and, we may say, inseparable relation to some pathological condition of the soft or hard tissues, resulting either from accident or disease. Indeed, the practice of conservative surgery involves so largely the application of the principles of medicine, that we almost lose sight of its manipulative features except as something accessary to curative or remedial measures.

In the practice of ophthalmic and aural surgery, the oculist and aurist deals, almost exclusively, with morbid conditions, and what there is of a manipulative character in the practice bears the same intimate and inseparable relation to medical treatment as that which obtains in respect to the practice of general surgery, and cuts scarcely any figure at all in the general estimate of requirements.

Having called your attention to these cardinal differences, it only remains, gentlemen to make some application of the fact. I need hardly say that, in calling your attention to these fundamental differences, there has been any purpose of detracting from the equal claims of dentistry upon the public regard, or from the important bearing of its practice upon the welfare of communities. Its creditable standing as one of the liberal professions is fully attested by its nearly half a score of colleges; by its numerous associations, national, state, district and local; by its multiplied and ably conducted journals; and by its text books, and minor works. I think it may be truthfully affirmed that, in all these respects, as well as in the character, intelligence, and general attainments of its professors, in the commensurativeness of its resources to the needs of the unfortunate and afflicted, and in the beneficent character of its ministrations, if it does not stand squarely in advance of other special departments of general medicine, it will at least compare favorably with them.

My chief purpose in considering the anomalous character in respect to the nature and demands of dental practice, is to account in some satisfactory manner for our present condition of isolation as a department of medicine, and to vindicate the policy of our present distinctive educational system, and the wisdom and forecast of the early pioneers who, by the inauguration of this system, declared dentistry a separate and independent professional organization.

There is that in the educational appliances of medical colleges which fully meets all the demands and requirements of the medical specialties. Under that system they may, by reason of the nature and requirements of their several departments, acquire complete and symmetrical development, because the system of medical instruction is fully adapted to, and commensurate with, all their needs. But no one who will stop to consider the phenomenal nature of the requirements of our calling will doubt for a moment but that had dentistry, like the other branches, been grafted upon the medical profession, and cultivated and practiced under the same educational limitations and restrictions, it not only would not have acquired any proper development, but would have been utterly and hopelessly dwarfed. The late Dr. Eleazer Parmly enunciated a truth which is worthy of all acceptance as an aphorism when he declared, more than thirty years ago: "I regard it as one of the wildest chimeras of the age to suppose for a moment that a practical education in the difficult art of dentistry can ever be attained in the class lecture rooms of the best medical college in the world."

It has doubtless often been a matter of curious speculation with many why dentistry, having relation to the study and treatment of important organs of the body, did not, like other special practices, have its origin within the body of the medical profession. A key to the solution of this interesting problem may be found, I think, in the fact that those who espoused it in the earlier part of the present century, recognizing as they undoubtedly did, that its practice necessarily embraced, and must always in any possible stage of future development embrace, an extended range of mechanical and manual processes and appliances, had an intelligent, and we may say intuitive, perception of the fact that there was nothing in the system of general medical education which could then, or ever afterward, meet in any sufficient degree the absolute and imperious demands of a practtice which, compared with the established medical specialties, was wholly and unchangeably phenomenal. The broad recognition of the peculiar needs of their calling was the great centrifugal force acting in obedience to a law of necessity which, as the art advanced, carried it farther and farther away from the medical profession, until its status as an independent and self-asserting organization became fixed in 1840 by the establishment of the first dental college at Baltimore empowered to grant special degrees.

This was the first institution of the kind in the world, and being itself wholly anomalous was consistent with the character of the theory and practice it was ordained to teach. Its establishment was a formal pronunciamento declaring in round terms to the medical profession that dentistry had decided to "go it alone," and was a deliberate and voluntary renunciation of all claims to recognition of it as a medical specialty proper by the medical profession. It was the critical point of departure where dentistry elected to become a separate and distinctive organization, rather than have its practice merged in that of general medicine, on equal terms with the recognized specialties. The wisdom of this choice has been fully vindicated in the marvelous progress of our profession since that time. It was a declaration of independence which should be annually commemorated by us with grateful hearts, for, in the fruits which it has borne, it has been to us as a profession what the declaration of 1776 has been to us as a nation. The inauguration of this pioneer school of dentistry was the grand nucleus from which has sprung, by gradual accretions, our present amplified system of professional education, symmetrical and rounded in in all its proportions, the crown and inspiration of American dentistry.

I have said that the founding of dental colleges, empowered by their charters to grant special degrees, was a voluntary renunciation of all claims to recognition of our calling as a medical specialty proper. Previous to the establishment of the Baltimore school, it would have been entirely practicable for its founders to have fixed the status of our department as a medical specialty by accepting the curriculum of the medical schools as a basis of dental education, with chairs for special instruction in our department. They were men generally not only of character, influence and liberal attainments, but many of them medical graduates, and nothing would have been easier than for them to have placed us as a department side by side with the recognized specialties. Indeed, such a policy was not only vehemently urged by a few at the time, but the experiment was actually tried in connection with the medical schools of that day, but the scheme was soon abandoned as wholly inadequate to meet the peculiar exigencies of dental practice, and many who were the first to take lectureships in the medical schools hastened to identify themselves with the "new departure" by accepting chairs in the dental college. Had the original plan been successfully inaugurated and enforced, we should to-day, as a department of medicine, occupy precisely the relation to the medical profession as general, aural and ophthalmic surgery. But the voluntary ignoring of the explicit requirement of a general medical education, which the acceptance of such a relation would have imposed, and the formal adoption of the partial system of medical education embraced in the curriculum of our dental colleges, with its appropriate and distinguishing badge of Doctor in Dental Surgery, placed us at once without the pale of the recognized medical specialties. This is the self-imposed attitude of dentistry to-day, and while it is entitled to be regarded as a veritable department of medicine, and is so generally accredited by the medical faculty, yet the formal recognition of it as such by any standard of requirements applicable to the other departments mentioned, can never consistently be accorded to it without abandonment of its specific and distinctive methods of collegiate instruction, and its accompanying conferment of special degrees.

There is probably no subject now before the profession engrossing so much of its attention as that of dental education. What, if any, ulterior purposes of innovation upon the established system of collegiate instruction may be contemplated by the agitation of this subject, is a matter which addresses itself to the serious and thoughtful consideration of all who are friendly to that system, and desire its perpetuation intact. If the recently awakened interest in this snbject contemplates only some supposed needful reforms in the working machinery of our dental colleges, or the rectification of any existing evils or shortcomings outside of these schools, we may all cross palms in mutual endeavors to provide the proper remedies. But if it is meant to force recognition of our calling as a medical specialty by such changes in the organic structure of these schools as shall seriously cripple their efficiency in respect to the adaptability of their methods of instruction to the peculiar needs of legitimate dentistry; or if it shall go farther and seek, through processes of disintegration, to ultimately subvert these institutions altogether, then it becomes the duty of the friends of our present system to rebuke, in unmeasured terms of disapprobation, all such revolutionary devices.

To be forewarned is to be forearmed. It does not require, I think, any very remarkable degree of penetration to discover in recent events and utterances a covert purpose of attaining for our department the status of an accepted medical specialty at all hazards. Much of this recent talk about the necessity of a broader medical culture is but the prologue to disorganizing schemes not only menacing the unity and integrity of the profession, but putting in peril our entire system of dental education. There are unquiet spirits who are prepared, at any cost to the established order of things, to bridge over the chasm which yawns between them and open recognition. This is a grave accusation, perhaps, but is not made, I think, without sufficient warrant. One phase of the disorganizing elements at work is discovered in a recent undisguised assault upon the unification of the departments of dental practice with a deliberate proposition of dismemberment.

Another, and more insidious, and therefore more dangerous, outcropping of disaffection in the ranks shows itself in the school which has fastened itself upon the medical department of the Harvard University. The organization of this parasitical body, with its miserable affectation of superior medical attainments implied in its degree of Doctor in Dental Medicine, is a virtual repudiation of the independent organizations which have conferred upon the dentists engaged in the enterprise all the professional character and distinction they enjoy. Of this educational barnacle, Dr. Arthur is pleased to say: "Harvard University, to its great credit, has lately created under its charter a 'School of Dentistry,' fully admitting by this action the claim of dentistry to the position for which we are contending." (That of a medical specialty.) Whether this action was voluntary upon the part of the university, as implied here, or the result of officious and persistent solicitation and importunity on the part of certain ambitious gentlemen whose voracious appetites for medical pabulem were unappeased by the limited rations doled out by the established institutions, remains a chapter of unwritten history. That the Harvard school is content to be received by the university medical faculty with something of that patronizing condescension usually accorded to "poor relations," or that it should be satisfied to shine by a reflected light in consideration of the distinction claimed for it by Dr. Arthur, is not a matter of any special concernment to us, except only so far as it indicates the animus which led to a departure from established methods of instruction. That man must indeed be dull of apprehension, I think, who does not discern in this movement a covert purpose of ultimate subversion of our present distinctive system of collegiate instruction, and the substitution of the medical curriculum. Let us see what warrant we have for this statement.

Dr. Robert Arthur, one of the foremost and most influential men in the profession, in an address before the last meeting of the Southern Dental Association, after an earnest appeal for a broader medical culture on the part of the dentist, says: "Where is this broad knowledge of the principles of medicine, and familiarity with the general features of disease to be obtained? From medical sources. If dentistry is a specialty of medicine it requires the study of medicine as much as ophthalmic surgery. Precisely the same necessity for it exists. 'The higher education, the broader culture of the physician,' is just what is needed at the present time by those who propose to engage in the practice of dentistry, to enable them to understand fully its requirements."

Just how dentists are to obtain this "broader medical culture of the physician," Dr. A. does not inform us. He is too clear-headed to suppose that it will ever be aecomplished by the introduction of a full corps of medical teachers into our dental colleges. This, even if it were practicable, would be to convert our dental into medical schools, with one or two chairs devoted to the teaching of the more practical departments. It would be precisely the same in effect as carrying our department into the medical colleges, and attaching chairs for special instruction. Economically, the latter would be much the better plan. If we maintain our present organization, and make a course in the medical schools a prerequisite of admission to the dental colleges, there will no longer be any need of chairs in the latter devoted to teaching the so-called higher branches, and the dental curriculum would contemplate only instruction in such departments of dental practice as would render the granting of the degree of Doctor in Dental Surgery too broad a farce to be tolerated.

It is but too apparent that much of this clamor for the broader medical culture of the physician will bear but one interpretation, and that is ultimate abandonment of our distinctive system of collegiate instruction and the acceptance of the medical curriculum as the basis of dental education. If any further evidence of this were wanting, it may be found in what a correspondent of the Philadelphia *Medical Times* has to say of the discussions of the Odontological Society of New York on the subject of dental education. As indicative of their temper and drift, he says:

"The subject of professional education and practice, which was discussed with unusual warmth, exhibited markedly the inclination entertained for a wider sphere of work, and portrayed plainly enough that the conversion of the dentist into the oral surgeon is only a question of time.

"The ranks of the common mother profession would receive a valuable addition in such gentlemen as compose the New York Odontological Society, and we most cordially unite with the speaker from your own city in trusting that the day is not distant when all shall be children of one Alma Mater, working in harmony and with a mutual interest for the common good of suffering humanity."

From all this it seems to me that sooner or later the issue must be made whether we are to fall back upon methods of instruction which the experience of the fathers in dentistry demonstrated a failure, or whether we shall stand in defense of a system which not only fully meets all the diversified and phenomenal requirements of practice, but which within the last half century has wrought a miracle of symmetrical development and growth in all its departments that has given renown to American dentistry and precedent distinction to its practitioners wherever the art and science of dentistry is known throughout the civilized world. To you. gentlemen, may be assigned some part in the final determination of this most important question.

I yield to no one in aspirations for a better acquaintance with the principles of medical science, but I desire such knowledge for its own sake, and as a helpful aid in the more intelligent discharge of my duties and responsibilities as a dentist. What we all, perhaps, most need is not so much a broader medical culture as a more exact and profound acquaintance with the medical branches already embraced in the curriculum of our dental colleges. I apprehend that there are no possible emergencies of dental practice which the right study of these branches, and a familiar acquaintance with the special textual and periodical literature of the profession, will not meet, and anything beyond these being a matter of accomplishment, any individual member of the profession is at liberty to so adorn himself if his time and inclinations prompt him. For one, I accepted dentistry nearly a quarter of a century ago for what it was then, and I esteem and honor it for what it is to-day, but more for its great beneficence and eminent usefulness than for any abstract quality of dignity or distinction that may attach to its practice. These will take care of themselves, we may rest assured. Consideration for our calling, both by the public and the medical faculty, will follow just in proportion as we shall practice it with right perceptions of the peculiar and phenomenal nature of its requirements. Whenever we loose sight of the exceptional aspects of our calling, and attempt to force it into positions and relations incompatible with the nature and demands of its practice, we at once lend ourselves to the work of weakening its influence, impairing its usefulness, and of circumscribing the grand possibilities of its future.

I thank you, gentlemen, for your patient attention to what I have had to say this evening upon subjects of mutual interest to us, and will close by expressing an earnest wish that you may realize the largest measure of succes in the pursuit of a calling which will afford you ample scope and opportunities tor the conjoined services of both hands and brain. We have the highest assurance that your preliminary training in the school which has this evening honored you with its indorsement will fitly qualify you for the creditable discharge of whatever duties the exigencies of practice may impose upon you. With repeated assurances of the best wishes of myself and others for your future health, happiness, and success in life, I bid you, gentlemen, good night.

